# The Potential of Photoacoustic Imaging in Detecting and Managing Complex Wounds

**DOI:** 10.34133/bmr.0206

**Published:** 2025-05-21

**Authors:** Haifeng Hu, Ruiyin Zeng, Longyu Du, Weixian Hu, Chuanlu Lin, Jiewen Liao, Chong Ding, Xudong Xie, Bobin Mi, Wu Zhou, Yun Sun, Faqi Cao, Guohui Liu

**Affiliations:** Department of Orthopedics, Union Hospital, Tongji Medical College, Huazhong University of Science and Technology, Wuhan 430022, China.

## Abstract

Photoacoustic imaging (PAI) is a promising emerging technology in biomedical imaging, particularly in wound healing. This review summarizes the applications of PAI in the detection and management of complex wounds, emphasizing its advantages in providing high-contrast, high-resolution deep tissue imaging. PAI integrates optical imaging’s high contrast with ultrasound’s deep penetration, facilitating the monitoring of vital physiological parameters like blood flow, oxygen saturation, and tissue regeneration in wounds. The review details the applications of PAI in monitoring wound pH, nerve repair, drug absorption, burn imaging, and infection-related wound assessment. It also explores the role of novel materials like carbon-based materials, nanorobots, and inorganic nanoparticles in enhancing PAI capabilities. Despite the technical challenges and limitations in clinical applications, PAI holds tremendous potential for wound healing monitoring. The review concludes by addressing the challenges and solutions for PAI, along with future development directions, to facilitate the transition of PAI technologies from experimental stages to clinical application.

## Introduction

The skin serves as the primary defense against chemical, physical, and pathogen threats while also contributing to immune function and water balance regulation [1]. Traumatic skin damage disrupts the body’s normal environment, affecting regular cell functions such as those of endothelial cells and fibroblasts across different skin layers. Consequently, wound healing, which seeks to restore normal skin functions, is a complex and highly regulated process [2,3]. This process involves cell migration, proliferation, differentiation, coordinated signaling pathways, and matrix remodeling [4]. Acute and chronic wounds pose substantial challenges in clinical practice, affecting patients, their families, and the public health system, and leading to considerable economic, social, and psychological burdens. Consequently, effective wound healing is crucial for patient recovery and the enhancement of their quality of life [5]. Wound assessment is crucial in wound healing. Accurate and timely detection helps healthcare professionals monitor the healing process [6], evaluate treatment effectiveness, and identify complications such as infection or excessive scarring early [7–9]. However, traditional methods of wound assessment have limitations [10]. For instance, visual inspection and manual measurement are not only subjective but also difficult to quantify and standardize [11], and they are susceptible to environmental influences [12–14]. Additionally, these methods usually cannot provide detailed information about the internal tissues of the wound, such as pH, blood flow [15], oxygen levels, and cellular activity, which are essential for evaluating wound healing [16,17].

The limitations of traditional wound assessment methods have led to the need for more advanced technologies [18]. Modern medical research has begun exploring the use of imaging technologies, biosensors, and other advanced methods for wound assessment [19]. These new technologies promise to provide more objective, accurate, and comprehensive information on wound healing, thereby improving patient treatment outcomes and prognosis.

**Fig. 1.**
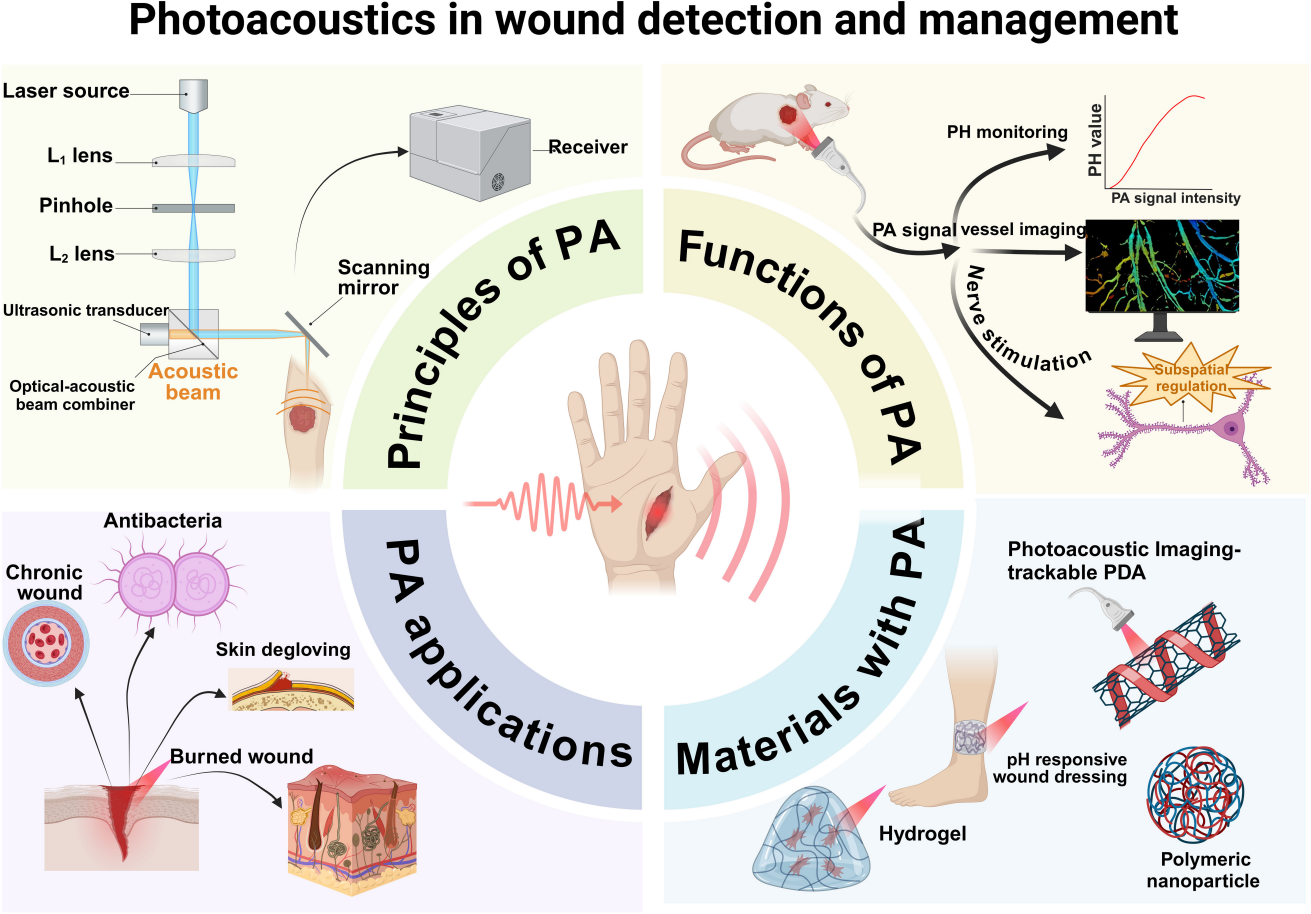
A brief introduction figure of PAI, including the principles of PA, the functions of PA, applications of PA, and newly invented materials with PA. Created in BioRender (Haifeng H, 2024; https://BioRender.com/l93f346).

In recent years, photoacoustic imaging (PAI) has received widespread attention due to its excellent performance in imaging depth, spatial resolution, and contrast between different tissues [20]. PAI is a hybrid technology that merges the benefits of optical and ultrasound imaging [21]. PAI, with its distinct mechanism, holds significant promise for biomedical research, particularly in wound healing. This system involves delivering pulsed or amplitude-modulated light energy to the target tissue. The tissue absorbs this light energy and converts it into heat, causing the tissue to undergo transient thermoelastic expansion and emit broadband ultrasonic waves [22–33]. These ultrasonic signals are received by the transducer and converted into visual image signals. This method integrates the high contrast and resolution of optical imaging with the deep tissue penetration of ultrasound. PAI in wound healing monitoring delivers detailed structural images of wound tissues and crucial diagnostic insights by evaluating functional parameters like blood oxygen levels and flow velocities [34–37]. PAI has been piloted in various systemic diseases and is progressively transitioning into clinical applications, offering a novel approach for disease screening, diagnosis, and longitudinal monitoring. In 2021, PAI received approval from the U.S. Food and Drug Administration (FDA), followed by its integration into the digital imaging and communications in medicine (DICOM) international standard in 2023. These milestones signify the evolution of PAI from experimental research to a clinically viable imaging modality [38]. This efficient, noninvasive imaging method is gradually becoming a powerful tool for wound healing assessment and treatment supervision (Fig. 1).

## Principle and Characteristics of PAI

Short light pulses absorbed by chromophores within biological tissues occur during irradiation. The absorption raises the chromophores’ temperature, emitting photoacoustic (PA) waves via a thermoelastic process. PA wave propagation on tissue surfaces reveals chromophore layer depth [39–41]. Wound sites typically exhibit vascular occlusion [37,42], while uninjured tissues generally maintain greater blood flow [43]. Pulsed light, selectively absorbed by blood, traverses damaged tissue with minimal loss and is absorbed by blood in healthy tissue layers, generating PA waves. This PA effect leads to hemoglobin expansion, generating a PA signal that comes as an acoustic pressure wave. Tissues at different depths will absorb pulse light, then generating a spectrum of PA signals that further facilitates the detailed mapping of the vascular conditions, oxygen saturation, and tissue composition. PA signals, detectable by a transducer positioned at the wound site [44,45], offer valuable information based on the wave propagation time to the tissue surface.

PAI merges the detailed optical contrast of optical imaging with the high-resolution characteristic of ultrasound imaging. Traditional optical imaging techniques offer high spatial resolution, but their effectiveness is limited to depths of around 1 to 2 mm due to photon absorption by complex tissue structures. Ultrasound imaging can provide sufficiently high spatial resolution, but it suffers from speckles and artifacts [20,46,47]. The emergence of PAI, coupled with continuous innovation in exogenous contrast agent materials (such as molecular probes, nanoparticles, and carbon-based materials), has significantly enhanced its sensitivity and specificity. Although the overall imaging depth is limited by the optical properties of tissues, we can still improve image reconstruction and tissue penetration by optimizing optical wavelengths or employing more advanced computational strategies. These advancements help overcome challenges like speckle and artifact formation, providing more robust tools for clinical applications. Moreover, studies have shown that subjects with darker skin exhibit increased PA signal at the skin surface, reduced signal penetration depth, and more pronounced streak artifacts, speckle, and clutter. This suggests a need for further clinical observations and discoveries [48].

**Fig. 2.**
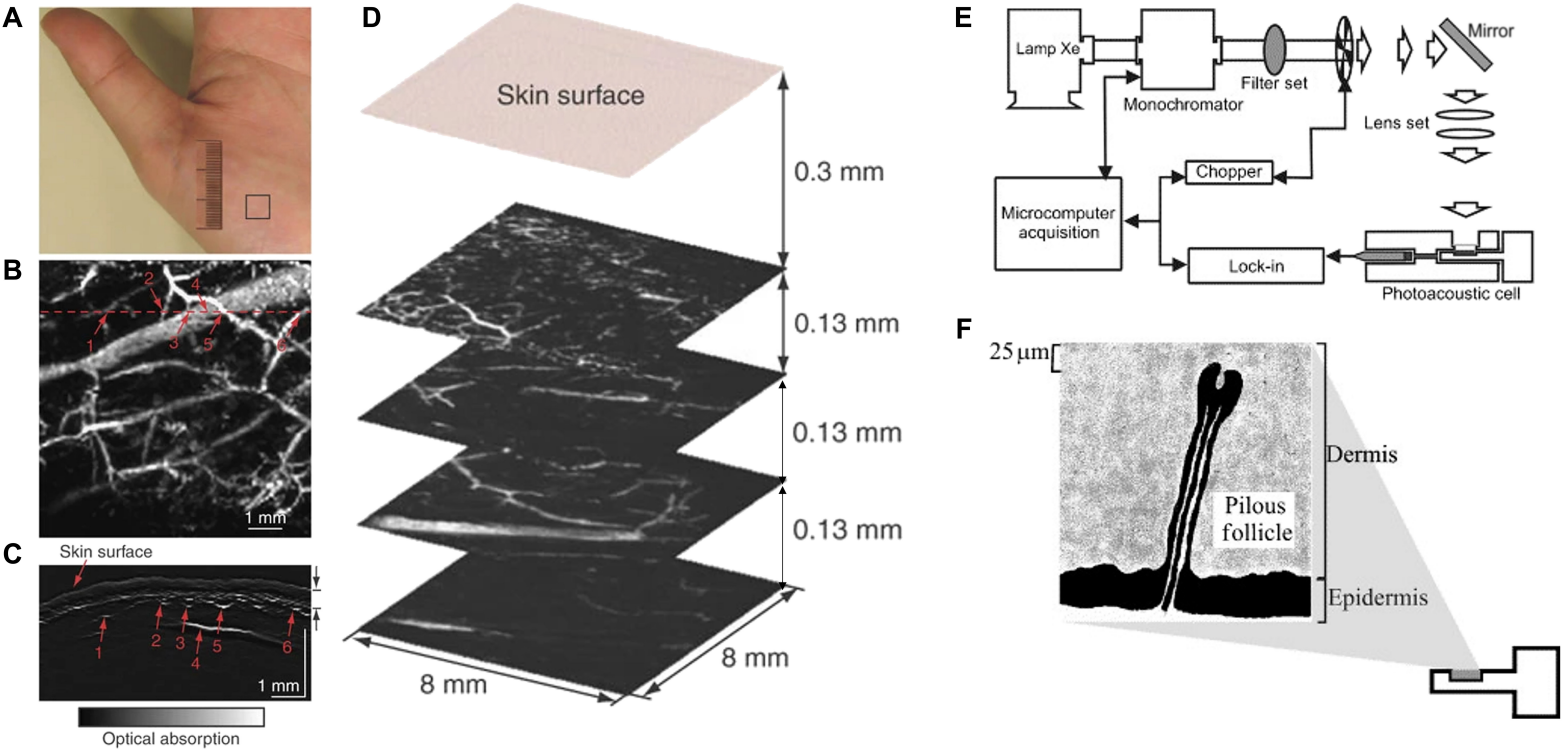
Illustrates the use of PAI for visualizing skin structures and details the setup of the PAI instrument. (A) Image of the photographed area. The black squares indicate the imaging areas. (B) MAP (microvasculature of the palm) representation of the PA signals beneath the stratum corneum layer, projected along the *z* axis. The B-scan image, located in the *z*–*x* plane at the dashed lines in (B), reveals the skin surface, stratum corneum, and blood vessels. (C) Numbers 1 to 6 correspond to the vessels in both the MAP and B-scan images. (D) Images of MAP from multiple layers parallel to the skin surface. (E) Illustration of the PA spectrometer. (F) Depiction of the skin sample within the PA chamber. Figures reprinted with authorization from [44]. © 2006, Springer Nature America Inc. Figures reprinted with permission from [73]. © 2007 Elsevier B.V. All rights reserved.

The conventional optical parametric oscillator (OPO) PA transducers, characterized by their bulkiness and limited mobility, pose a significant challenge to clinical procedures. Notably, emerging transducers exhibit favorable attributes such as compactness and portability, complemented by their capacity to accept a spectrum of PA signals suitable for detection tasks. Various commercial options, including the hockey-stick transducer (ATL CL15-7, Philips) [49], handheld imaging scanners [50], and the PAI pens [51], represent promising avenues that may potentially inspire development of innovative imaging tools (Fig. 2).

## Application of PAI in Wound Detection

### pH monitoring to aid in chronic wound healing

In chronic wounds, changes in the pH value and metabolites of the wound exudate can provide clinicians with valuable information about the wound’s status [52]. The pH value serves as an important indirect reference for evaluating oxygen transport, protease activity, angiogenesis, and bacterial infection [53–55]. Under normal conditions, an acute wound maintains an acidic pH value (pH 4 to 6). The pH value of a wound initially increases at the onset of injury. In acute wounds, the pH level rapidly reverts to the normal acidic range. Proteases are generally more active under mildly acidic conditions (pH 6.5), which aids in promoting the expression of proteins that facilitate wound healing. However, chronic wounds maintain an alkaline (pH 7.4 to 9) over extended periods [56,57], making pH monitoring crucial for effective management [58]. Guo et al. [59] suggest that the PA method could effectively monitor the pH of such wounds. Guo’s team developed a pH-responsive hydrogel dressing. Integrating pH-sensitive molecules with superior PA capabilities into the hydrogel enabled rapid, noninvasive wound pH detection. Monitoring pH assists physicians in assessing wound conditions and evaluating the effectiveness of dressings, aiding in the decision of whether surgical intervention or the use of antimicrobial and anti-inflammatory medications is necessary [60,61].

### Monitoring nerve repair

Studies have shown that low-intensity pulsed ultrasound can enhance peripheral nerve regeneration and stimulate the nervous system. PA stimulation, with its high spatial resolution, has been proven to be a precise method for nerve stimulation, effectively promoting nerve regeneration and offering a novel approach for neural repair in wound healing. In the PA process, pulsed light targets absorbers, inducing rapid heating and thermal expansion that produce broadband ultrasonic acoustic waves. These acoustic waves stimulate nerve tissues, prompting them to respond to the stimulation, thereby enhancing synaptic growth and facilitating functional regeneration [62–64]. The team’s research findings indicate that by using novel probe materials combined with PA technology, it is possible to achieve submillimeter spatial resolution (100 μm)-level neuromodulation, overcoming the limitations of traditional transducer-based ultrasound therapy [64–67].

### Monitoring drug’s PA signal in wound

**Fig. 3.**
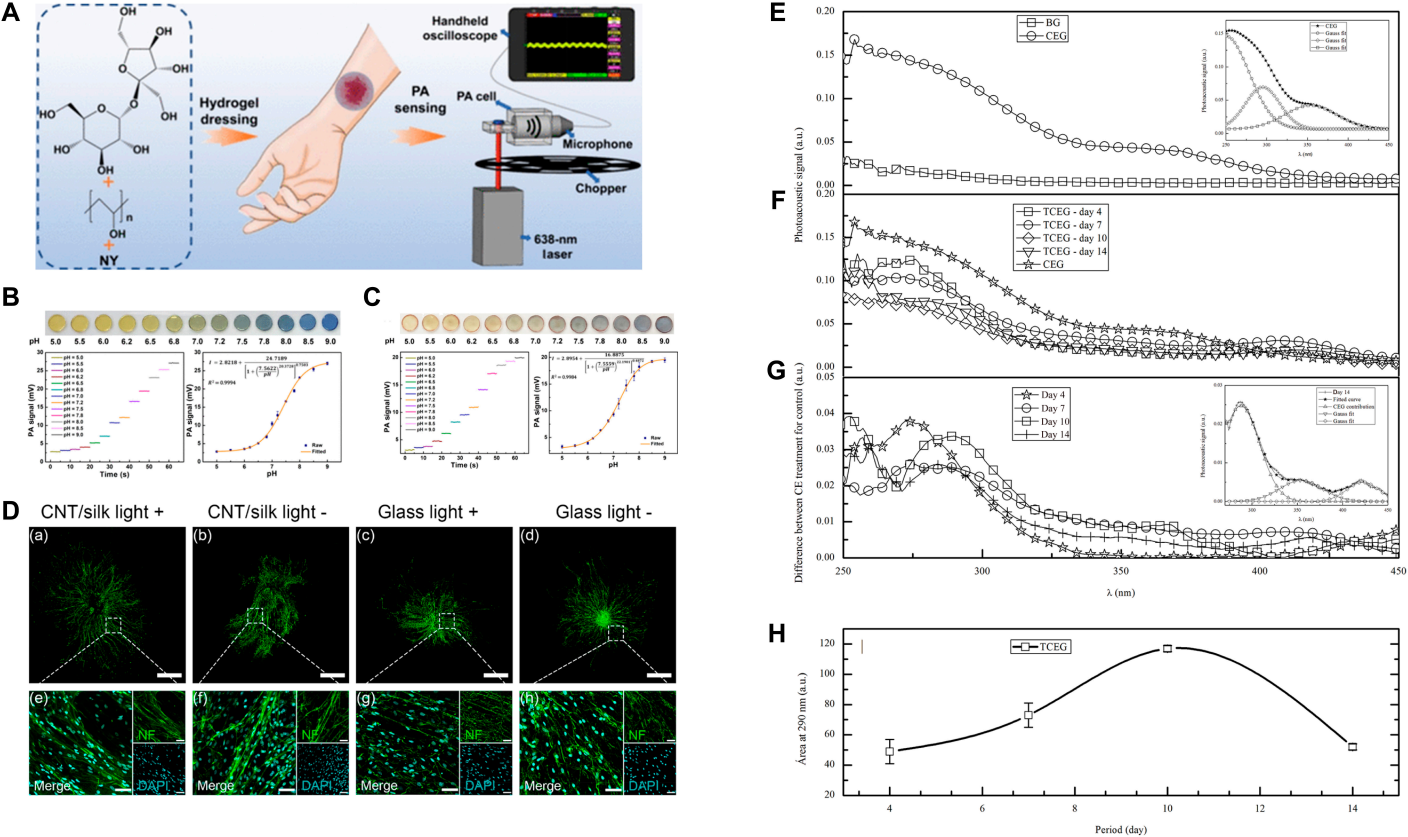
Multiple aspects of the application of PA technology in wound healing. (A) The portable PA device, integrated with a PVA/Suc/NY hydrogel dressing, is designed for point-of-care wound pH testing. Images and PA signals of PVA/Suc/NY hydrogels in phosphate-buffered saline solutions with varying pH levels are included. (B) Correlation between PA signal and pH level. (C) Photographs and PA signals of PVA/Suc/NY hydrogels with varying pH levels of human blood, illustrating the correlation between PA signals and pH values. (D) The findings indicate that PA techniques can effectively promote the growth of dorsal root ganglion synapses (DRGs) in rat embryos. The study includes (E) PA spectra of base gel (BG) and gel with 1% CE (CEG), with a Gauss fit of crude extract (CE) absorption bands in the inset; (F) PA spectra of wound treated with CEG (TCEG) over a 14-d treatment period; (G) the difference between TCEG and control dermal spectra, with a Gauss fit of CEG absorption bands in the inset; and (H) the permeation behavior area of the absorption band at 290 nm (control: wound treated with BG). This content is reproduced under the Creative Commons Attribution 4.0 International License. Figures are reproduced with permission from [64], © 2022 American Chemical Society. Figures are reproduced with permission from [59], © 2024 American Chemical Society. Figures are reproduced with permission from [68], © 2016 Bueno et al.

PA monitoring can also be utilized to track the drug’s PA signal. For instance, the team led by Bueno has employed PA spectroscopy (PAS) to compare the PA signal of wounds before and after drug administration to evaluate drug permeability [68,69]. By leveraging PAI’s high sensitivity and spatial resolution, researchers can obtain real-time, noninvasive insights into the drug’s distribution and diffusion within wound tissues [69–73]. Such capabilities are critical in optimizing therapeutic strategies, ensuring effective drug delivery, and ultimately enhancing wound healing outcomes. Integrating PA monitoring with therapeutics provides a robust framework for wound management (Fig. 3).

### Burn wound imaging

**Fig. 4.**
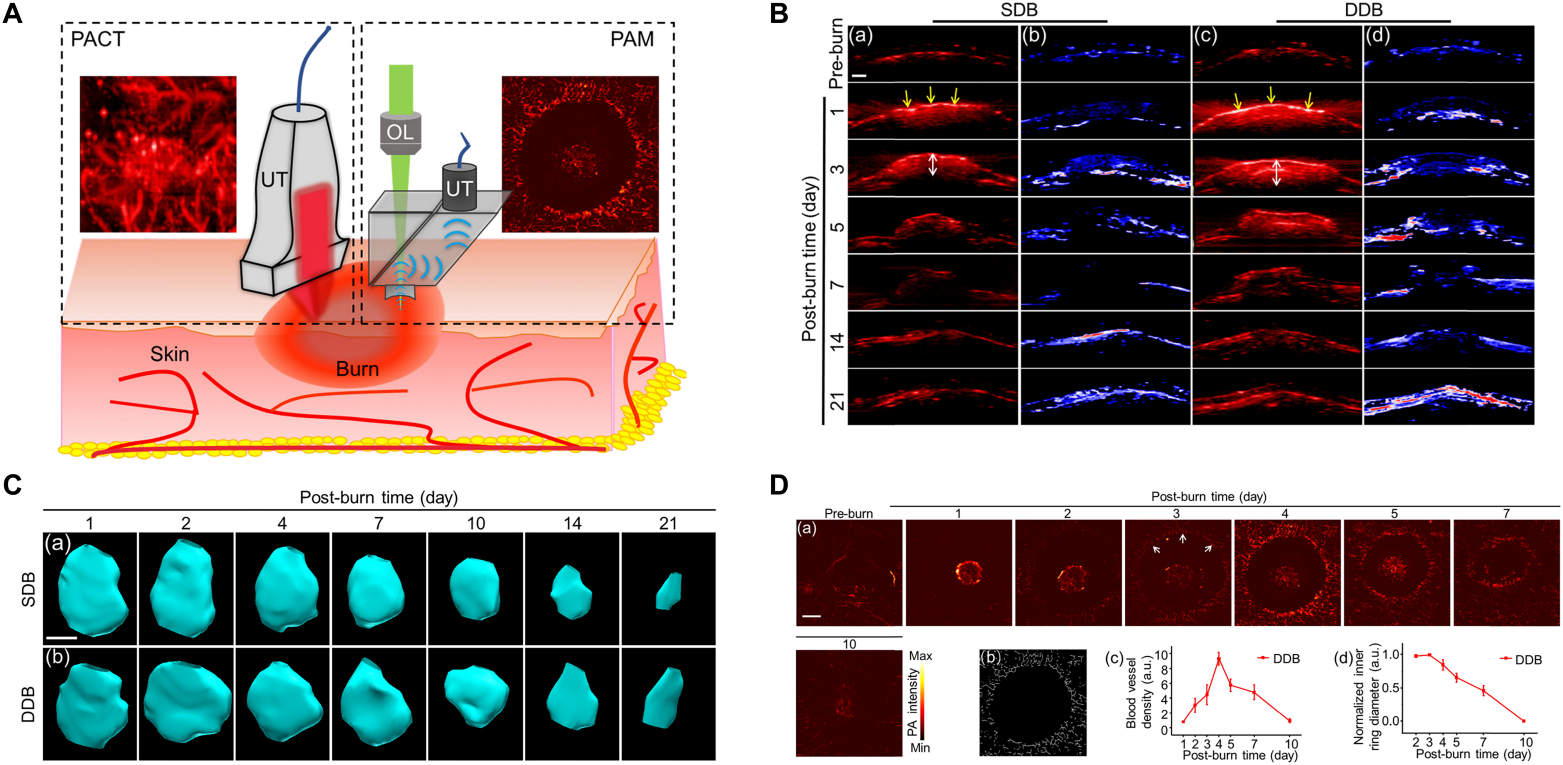
The use of dual-scale PAI to evaluate the progression and features of superficial dermal burn (SDB) and deep dermal burn (DDB). (A) Illustration of dual-scale PAI applied to skin burn injuries. (B) Images of burn injuries captured through cross-sectional PACT. Cross-sectional PA structural images illustrate SDB and DDB injuries at selected time points. Yellow arrows indicate the eschar on the skin surface post-thermal damage, while white double arrows show the burn wound depth. Cross-sectional SO_2_ images of (b) SDB and (d) DDB injuries are presented at selected time intervals. (C) The volume of burn injuries decreases over time. 3D maps approximating the reconstructed burn areas are provided for both (a) SDB and (b) DDB. (D) (a) PAM imaging of burn injury skin surfaces. High-resolution PAM images of DDB captured at various time points with a 1-mm scale bar. Blood vessel signals are strongest on day 4 and decrease after 10 d. The hyperemic ring is indicated by white arrows. (b) Vessel structure illustration extracted via a skeletonization algorithm at day 4. (c) Relative blood vessel density of (a). (d) Normalized diameter of the hyperemic ring's interior. This content is reproduced under the Creative Commons Attribution 4.0 International License [80], © 2019 Optical Society of America. According to the OSA Open Access Publishing Agreement.

PA technology can also aid in the monitoring and recovery of burn wounds. Clinical assessment of burns depends on 2 critical factors: burn depth and area [74]. Clinically, the judgment of burn depth mainly relies on visual inspection or needle probing tests, which are largely limited by the surgeon’s experience [75–77]. PAI can achieve deeper imaging depths while maintaining high spatial resolution [78], thereby providing detailed information about the vascular tissues in the deeper layers of burn wounds [79]. Wu’s team utilized PA computed tomography (PACT) and PA microscopy (PAM) to monitor and assess burn wounds. PACT delivers 3-dimensional (3D) structural and functional data of the burn site, whereas PAM provides high-resolution images of vascular alterations. Imaging the hyperemia ring, which marks the boundary of thermal damage on the skin, aids in understanding the healing process of burn wounds [80–84]. This innovative application of PAI technology could offer fresh perspectives for diagnosing and treating burns (Fig. 4).

### Monitoring and imaging of infected wounds

**Fig. 5.**
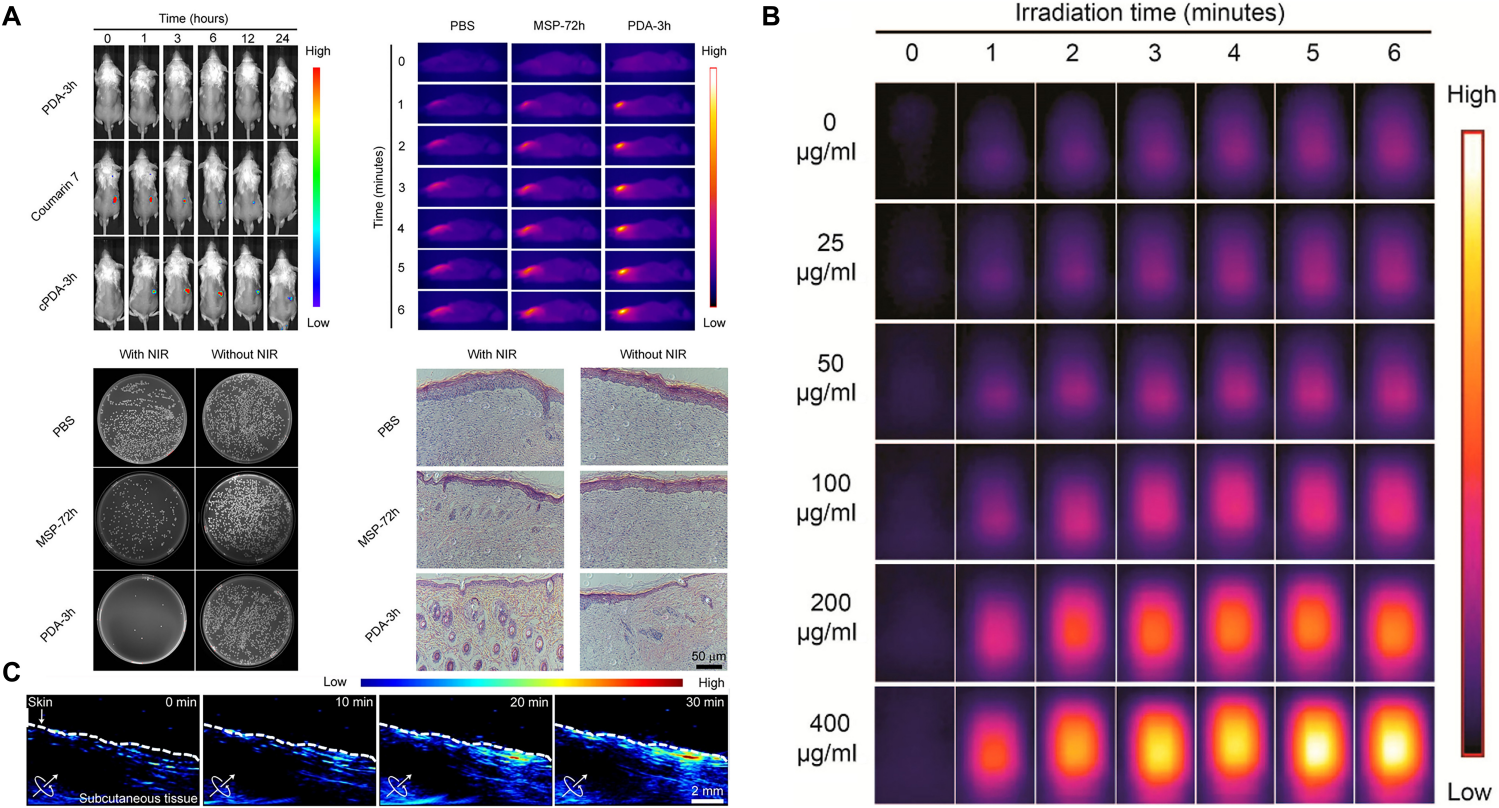
The use of PDA-MSP in PAI and photothermal therapy for treating bacterial infections. (A) The images comprise in vivo fluorescence sequences and thermal images of subcutaneous tissues in Balb/c mice infected with multidrug-resistant *Klebsiella pneumoniae* (MDR KP) under various treatments, bacterial culture plates from infected sites, and histological micrographs of tissues collected after treatment. (B) Assessment of the photothermal effect, PA signal, and image tracking capability. Thermal images over time of PDA-3h samples (μg/ml). Images were taken every minute. (C) Evaluation of PA image tracking and photothermal therapy in MDR KP-infected subcutaneous tissues of Balb/c mice. Time-lapse PA images were acquired every 10 min. Scale bar, 50 μm. Figures reprinted with permission from [94]. © 2020 American Chemical Society.

Antibiotic resistance has long been a vexing issue in the clinical treatment of bacterial infections. Prolonged use of antibiotics in medical treatment not only risks damaging the body’s immune system but also may lead to the emergence of multidrug-resistant superbugs [85,86]. Consequently, the exploration of safe and effective non-antibiotic therapies has become a focal point in current research [87]. Research conducted recently indicates that antibacterial photodynamic therapy (aPDT) might partially substitute antibiotics in treating infected wounds, with several advantages: (a) It is minimally invasive; (b) it offers a broad spectrum of antibacterial activity; and (c) in theory, resistance to antibiotics does not exist [88–90]. Numerous research teams have endeavored to apply aPDT for combating bacterial infections. However, before clinical translation can be achieved, challenges such as precise navigation, in vivo imaging, and real-time motion tracking still require effective solutions [91–93]. On this basis, many teams have made groundbreaking advancements. Xie et al. [94] described a magnetic microswimmer featuring a polydopamine (PDA) coating, consisting of a magnetized spirulina (MSP) matrix with a PDA surface. The PDA coating on the surface improves the MSP’s PA signal and photothermal effects. Additionally, fluorescent probes targeting bacterial surface antigens are added to the PDA surface. When the magnetic microswimmer triggers specific signals in its surroundings, these probes are released from the PDA, enabling an on–off fluorescence diagnostic mechanism. This innovation makes the integration of PAI tracking with photodynamic therapy possible. The advancements in photodynamic therapy, combined with innovative technologies like PAI, bring significant promise for enhancing the treatment of infected wounds. Through precise visualization and targeted therapy, there is a potential for substantial improvements in clinical outcomes in managing wound infections [95] (Fig. 5).

### Monitoring of vascular density

**Fig. 6.**
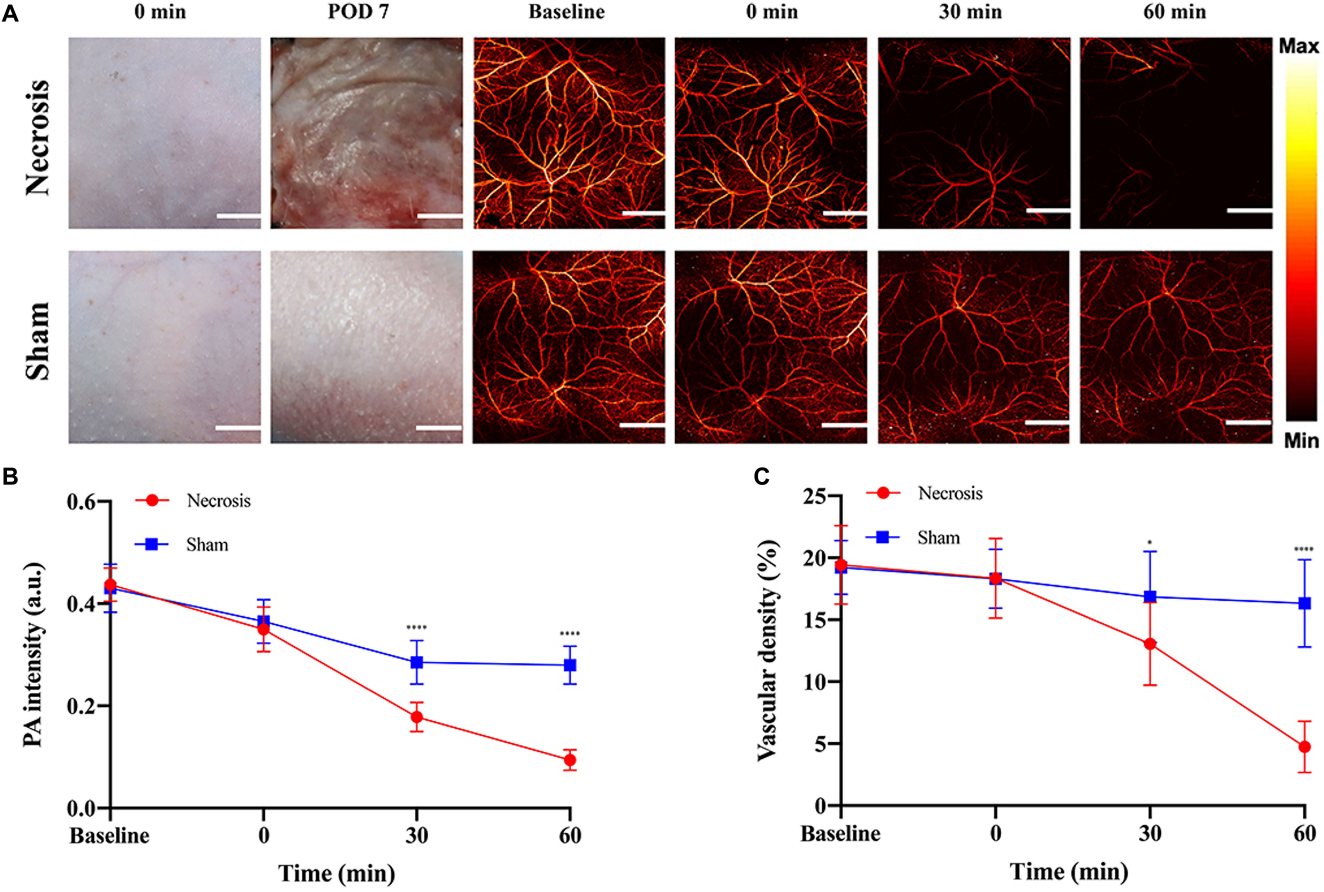
Demonstrates that PAI can monitor skin necrosis following skin avulsion. (A) Comparison of clinical outcomes on postoperative day 7 and PAM imaging at various time points in the region of interest between the avulsed hindlimb flap group and the sham group. (B) Quantitative analysis of PA intensity in the region of interest, as detected by PAM, comparing the Necrosis and Sham groups. (C) Quantitative analysis of vascular density in the region of interest, as detected by PAM, was conducted to compare the Necrosis and Sham groups. This content is reproduced under the Creative Commons Attribution 4.0 International License [99]. Copyright © 2021 The Author(s).

Skin degloving injuries, also known as Morel–Lavallée lesions, are associated with a high mortality rate and present a formidable surgical challenge in emergency situations [96–98]. PAM was applied to avulsion wounds to assess changes in vascular density and PA intensity, thereby evaluating the necrosis degree of the avulsion flap. PAI provides a promising approach for managing complex injuries by delivering high-resolution, real-time images of damaged tissues. This enables surgeons to accurately assess injury extent, monitor healing, and evaluate therapeutic efficacy, thereby allowing for better selection of therapeutic approaches. As a noninvasive technique, PAI combines optical and ultrasound imaging advantages, facilitating detailed visualization of both superficial and deep tissue structures [99] (Fig. 6).

Additionally, Hariri’s team demonstrated that the ability of PAI to detect early tissue damage and vascular ischemia provides valuable clinical application potential for staging pressure ulcers and diabetic foot ulcers [100]. As previously mentioned, the capability of PAM for microvascular imaging provides a clearer view during complex surgeries and can also obtain detailed images of vascular structures and functions in the brain, aiding surgeons in improving clinical outcomes [101].

### Relevant human subject data

PAI has been employed by several research teams to conduct small cohort and case studies. One team utilized PAI technology to assess the blood oxygen saturation in ischemic chronic wounds and mapped the vascular system of these wounds through PAI, providing more precise information for decisions regarding amputation surgery [57]. Additionally, other teams have applied PA techniques for functional imaging of anatomical structures such as tendons in human subjects, aiming to offer more useful imaging tools for clinical applications [102]. Similarly, in the field of burn wounds, teams have reported the use of various imaging acquisition systems in human subjects with burn injuries, further demonstrating the reliability of PAI in clinical settings [103]. Furthermore, some research teams have employed PAI technology to evaluate the levels of CH4 in the exhaled breath of severely injured patients as a means of assessing the extent of trauma [104]. Despite the wealth of imaging information obtained from numerous subject data, there remains a lack of standardized regulations regarding instrumentation, imaging parameters, and appropriate imaging time points in these studies, indicating that further efforts are needed to facilitate the clinical implementation of PAI technology.

## Applications of Novel Materials in PAI

The emergence of novel materials in the field of PA imaging presents new possibilities for enhancing the capability and efficacy of this innovative diagnostic technology. These advancements are particularly crucial in overcoming current limitations in imaging depth and resolution. Novel materials, when combined with PAI, address the limitations of traditional endogenous contrast agents like hemoglobin and melanin. Consequently, there is a substantial research focus on developing and integrating advanced materials to enhance the specificity and sensitivity of PAI, particularly in wound healing. These materials include hydrogels [59], carbon nanotubes (CNTs) [105–107], reduced graphene oxide [108,109], and semiconducting polymer nanoparticles (SPNs) [110,111], among others.

### Carbon-based materials

In PAI for wound healing, carbon-based materials offer distinct benefits. For example, certain studies have utilized a nanocomposite method to integrate CNTs into silk for addressing wounds associated with nerve stimulation and regeneration [64]. The CNT/silk material utilizes a silk matrix for structural support in tissue growth [112], while embedded CNTs convert pulsed near-infrared (NIR) light into acoustic and thermal energy, making it suitable for PA applications that promote neural growth [113]. Silk fibroin is chosen as the matrix due to its FDA-approved biocompatibility and documented support for neuronal adhesion and function.

A research team has demonstrated the use of the commercial pH indicator Nitrazine Yellow (NY) for its promising PA capabilities. NY exhibits excellent PA properties in both acidic and alkaline environments, allowing it to indicate environmental pH through PA effects [114]. When incorporated into poly(vinyl alcohol)/sucrose (PVA/Suc) hydrogels, known for their superior biochemical and mechanical properties [115,116], it enables the creation of a wearable PA pH sensor with exceptional PA performance. This hydrogel patch can be used for pH monitoring in chronic wounds, facilitating real-time assessment of wound healing and timely treatment adjustments.

### Nanorobots

Nanorobots hold great promise for controlled and precise navigation in areas of the body that are hard to access. In the last 10 years, wireless, minimally invasive biodevices have been widely investigated for clinical applications [117,118]. Magnetic microswimmer nanorobots with PDA coatings are an innovative material in wound healing, utilizing PAI. These nanorobots consist of an MSP matrix coated with PDA. PDA demonstrates a strong affinity for PA signals, effectively inducing photothermal effects [119–121]. When combined with the magnetic microswimmer nanorobots, which possess excellent biocompatibility, high precision control, and the ability to penetrate deep tissues, these materials enable precise treatment of bacterial-infected wounds under the guidance of PAI [122]. They operate through various mechanisms, including magnetic actuation and photothermal therapy.

### Inorganic nanoparticles

Semiconductor polymer nanoparticles (SPN) exhibit strong near-infrared absorption and photostability, enabling precise imaging and drug delivery to deep wound layers when paired with targeting molecules [123–125]. Surface modifications can control their distribution and metabolism, enhancing their wound healing applications [126]. Advances in material science are expected to introduce more high-performance materials for wound healing guided by PAI, leading to breakthroughs in wound repair (Fig. S1).

### Mn-single-atom nano-multizyme

In carbon-based life forms, metal ion-dependent enzymes also enable research teams to design more materials with stronger PA effects while simulating various enzyme activities to achieve more biological functions. In the nanozymes designed by the Zhang team, this new material exhibits the synergistic effect of photothermal therapy and multiple enzymes to catalyze chemotherapy for cancer, enabling PAI to guide treatment implementation by revealing biological distribution dynamics and in situ monitoring [127]. This technology, which combines multifunctional enzyme properties with enhanced photothermal therapy effects, can also be applied in the treatment of wound infections and other types of infections, providing new technical ideas for wound healing.

## Conclusion

Advancements in PAI offer promising new perspectives for the diagnosis and treatment of wounds. During wound healing, it is crucial to assess blood flow, oxygen saturation, and the extent of new tissue formation. Traditional imaging techniques are often limited by imaging resolution and tissue penetration depth. PAI merges optical and ultrasound advantages, offering high-contrast, high-resolution images that penetrate deeper tissue layers. PAI systems have shown substantial clinical value in real-time monitoring and early diagnosis of diabetic foot ulcers, burn wounds, and both acute and chronic wounds. Ultimately, utilizing PA technology for wound healing assessment and treatment aims to improve patient outcomes. These advancements require ongoing interdisciplinary collaboration, supported by continual innovation in materials science, with the goal of transitioning PAI from experimental stages to routine clinical practice.

## Data Availability

Data sharing is not applicable for this article as no datasets were generated or analyzed during the current study.

## References

[1] Sharifiaghdam M, Shaabani E, Faridi-Majidi R, De Smedt SC, Braeckmans K, Fraire JC. Macrophages as a therapeutic target to promote diabetic wound healing. Mol Ther. 2022;30(9):2891–2908. 35918892 10.1016/j.ymthe.2022.07.016 PMC9482022

[2] Farahani M, Shafiee A. Wound healing: From passive to smart dressings. Adv Healthc Mater. 2021;10(16):e2100477. 34174163 10.1002/adhm.202100477

[3] Nguyen AV, Soulika AM. The dynamics of the skin’s immune system. Int J Mol Sci. 2019;20(8):1811. 31013709 10.3390/ijms20081811 PMC6515324

[4] Raziyeva K, Kim Y, Zharkinbekov Z, Kassymbek K, Jimi S, Saparov A. Immunology of acute and chronic wound healing. Biomol Ther. 2021;11(5):700. 10.3390/biom11050700 PMC8150999 34066746

[5] Li S, Mohamedi AH, Senkowsky J, Nair A, Tang L. Imaging in chronic wound diagnostics. Adv Wound Care. 2020;9(5):245–263. 10.1089/wound.2019.0967 PMC7099416 32226649

[6] Freedman BR, Hwang C, Talbot S, Hibler B, Matoori S, Mooney DJ. Breakthrough treatments for accelerated wound healing. Sci Adv. 2023;9(20):eade7007. 37196080 10.1126/sciadv.ade7007 PMC10191440

[7] Gurtner GC, Werner S, Barrandon Y, Longaker MT. Wound repair and regeneration. Nature. 2008;453(7193):314–321. 18480812 10.1038/nature07039

[8] Schreml S, Szeimies R-M, Prantl L, Landthaler M, Babilas P. Wound healing in the 21st century. J Am Acad Dermatol. 2010;63(5):866–881. 20576319 10.1016/j.jaad.2009.10.048

[9] Nagle SM, Stevens KA, Wilbraham SC. *Wound assessment. StatPearls*. Treasure Island (FL): StatPearls Publishing; 2024. 29489199

[10] Joorabloo A, Liu T. Smart theranostics for wound monitoring and therapy. Adv Colloid Interf Sci. 2024;330: Article 103207. 10.1016/j.cis.2024.103207 38843699

[11] Kingsley A. The wound infection continuum and its application to clinical practice. Ostomy Wound Manage. 2003;49(7A Suppl):1–7. 12883156

[12] Wang Y, Guo M, He B, Gao B. Intelligent patches for wound management: In situ sensing and treatment. Anal Chem. 2021;93(11):4687–4696. 33715353 10.1021/acs.analchem.0c04956

[13] Dargaville TR, Farrugia BL, Broadbent JA, Pace S, Upton Z, Voelcker NH. Sensors and imaging for wound healing: A review. Biosens Bioelectron. 2013;41:30–42. 23058663 10.1016/j.bios.2012.09.029

[14] Enoch S, Harding K. Wound bed preparation: The science behind the removal of barriers to healing. Wounds. 2003;15(7):213–229.

[15] Fernández-Ruiz I. Photoacoustic method enables deep imaging of blood flow. Nat Rev Cardiol. 2024;21(2):72. 10.1038/s41569-023-00985-w 38102479

[16] Eskilson O, Zattarin E, Berglund L, Oksman K, Hanna K, Rakar J, Sivlér P, Skog M, Rinklake I, Shamasha R, et al. Nanocellulose composite wound dressings for real-time pH wound monitoring. Mater Today Bio. 2023;19: Article 100574. 10.1016/j.mtbio.2023.100574 PMC9958357 36852226

[17] Schneider LA, Korber A, Grabbe S, Dissemond J. Influence of pH on wound-healing: A new perspective for wound-therapy? Arch Dermatol Res. 2007;298(9):413–420. 17091276 10.1007/s00403-006-0713-x

[18] Rogers LC, Bevilacqua NJ, Armstrong DG, Andros G. Digital planimetry results in more accurate wound measurements: A comparison to standard ruler measurements. J Diabetes Sci Technol. 2010;4(4):799–802. 20663440 10.1177/193229681000400405 PMC2909508

[19] Peti-Peterdi J, Kidokoro K, Riquier-Brison A. Novel in vivo techniques to visualize kidney anatomy and function. Kidney Int. 2015;88(1):44–51. 25738253 10.1038/ki.2015.65 PMC4490063

[20] Wang LV, Hu S. Photoacoustic tomography: In vivo imaging from organelles to organs. Science. 2012;335(6075):1458–1462. 22442475 10.1126/science.1216210 PMC3322413

[21] Xu M, Wang LV. Photoacoustic imaging in biomedicine. Rev Sci Instrum. 2006;77(4):041101.

[22] Jeon S, Kim J, Lee D, Baik JW, Kim C. Review on practical photoacoustic microscopy. Photoacoustics. 2019;15:100141. 31463194 10.1016/j.pacs.2019.100141 PMC6710377

[23] Mallidi S, Luke GP, Emelianov S. Photoacoustic imaging in cancer detection, diagnosis, and treatment guidance. Trends Biotechnol. 2011;29(5):213–221. 21324541 10.1016/j.tibtech.2011.01.006 PMC3080445

[24] Hu S, Wang LV. Photoacoustic imaging and characterization of the microvasculature. J Biomed Opt. 2010;15(1):011101. 20210427 10.1117/1.3281673 PMC2821418

[25] Emelianov SY, Li P-C, O’Donnell M. Photoacoustics for molecular imaging and therapy. Phys Today. 2009;62(8):34–39. 20523758 10.1063/1.3141939 PMC2879661

[26] Tam AC. Applications of photoacoustic sensing techniques. Rev Mod Phys. 1986;58(2):381–431.

[27] Wang S, Lin J, Wang T, Chen X, Huang P. Recent advances in photoacoustic imaging for deep-tissue biomedical applications. Theranostics. 2016;6(13):2394–2413. 27877243 10.7150/thno.16715 PMC5118603

[28] Kratkiewicz K, Manwar R, Zhou Y, Mozaffarzadeh M, Avanaki K. Technical considerations in the Verasonics research ultrasound platform for developing a photoacoustic imaging system. Biomed Opt Express. 2021;12(2):1050–1084. 33680559 10.1364/BOE.415481 PMC7901326

[29] Manwar R, Hosseinzadeh M, Hariri A, Kratkiewicz K, Noei S, Avanaki MRN. Photoacoustic signal enhancement: Towards utilization of low energy laser diodes in real-time photoacoustic imaging. Sensors. 2018;18(10):3498. 30336570 10.3390/s18103498 PMC6209994

[30] Mahmoodkalayeh S, Jooya HZ, Hariri A, Zhou Y, Xu Q, Ansari MA, Avanaki MRN. Low temperature-mediated enhancement of photoacoustic imaging depth. Sci Rep. 2018;8(1):4873. 29559653 10.1038/s41598-018-22898-2 PMC5861112

[31] Manwar R, Kratkiewicz K, Avanaki K. Investigation of the effect of the skull in transcranial photoacoustic imaging: A preliminary ex vivo study. Sensors. 2020;20(15):4189. 32731449 10.3390/s20154189 PMC7435985

[32] Nasiriavanaki M, Xia J, Wan H, Bauer AQ, Culver JP, Wang LV. High-resolution photoacoustic tomography of resting-state functional connectivity in the mouse brain. Proc Natl Acad Sci USA. 2014;111(1):21–26. 24367107 10.1073/pnas.1311868111 PMC3890828

[33] Shin S, Kim CH, Son S, Lee JA, Kwon S, You DG, Lee J, Kim J, Jo D-G, Ko H, et al. PEDF-enriched extracellular vesicle for vessel normalization to potentiate immune checkpoint blockade therapy. Biomater Res. 2024;28:0068. 39355307 10.34133/bmr.0068 PMC11443973

[34] Attia ABE, Balasundaram G, Moothanchery M, Dinish US, Bi R, Ntziachristos V, Olivo M. A review of clinical photoacoustic imaging: Current and future trends. Photoacoustics. 2019;16: Article 100144. 31871888 10.1016/j.pacs.2019.100144 PMC6911900

[35] Li M, Tang Y, Yao J. Photoacoustic tomography of blood oxygenation: A mini review. Photoacoustics. 2018;10:65–73. 29988848 10.1016/j.pacs.2018.05.001 PMC6033062

[36] Mantri Y, Dorobek TR, Tsujimoto J, Penny WF, Garimella PS, Jokerst JV. Monitoring peripheral hemodynamic response to changes in blood pressure via photoacoustic imaging. Photoacoustics. 2022;26: Article 100345. 35295617 10.1016/j.pacs.2022.100345 PMC8918860

[37] Mantri Y, Tsujimoto J, Donovan B, Fernandes CC, Garimella PS, Penny WF, Anderson CA, Jokerst JV. Photoacoustic monitoring of angiogenesis predicts response to therapy in healing wounds. Wound Repair Regen. 2022;30(2):258–267. 34985822 10.1111/wrr.12992 PMC8897271

[38] Park J, Choi S, Knieling F, Clingman B, Bohndiek S, Wang LV, Kim C. Clinical translation of photoacoustic imaging. Nat Rev Bioeng. 2025;3:193–212.

[39] Paul DW, Ghassemi P, Ramella-Roman JC, Prindeze NJ, Moffatt LT, Alkhalil A, Shupp JW. Noninvasive imaging technologies for cutaneous wound assessment: A review. Wound Repair Regen. 2015;23(2):149–162. 25832563 10.1111/wrr.12262

[40] Sato S, Yamazaki M, Saitoh D, Tsuda H, Okada Y, Obara M, Ashida H. Photoacoustic diagnosis of burns in rats. J Trauma Acute Care Surg. 2005;59(6):1450. 10.1097/01.ta.0000197389.94466.04 16394921

[41] Yamazaki M, Sato S, Saitoh D, Okada Y, Ashida H, Obara M. Photoacoustic monitoring of neovascularities in grafted skin. Lasers Surg Med. 2006;38(3):235–239. 16392144 10.1002/lsm.20272

[42] Petri M, Stoffels I, Jose J, Leyh J, Schulz A, Dissemond J, Schadendorf D, Klode J. Photoacoustic imaging of real-time oxygen changes in chronic leg ulcers after topical application of a haemoglobin spray: A pilot study. J Wound Care. 2016;25(2):9–91. 10.12968/jowc.2016.25.2.87 26878301

[43] Rebling J, Ben-Yehuda Greenwald M, Wietecha M, Werner S, Razansky D. Long-term imaging of wound angiogenesis with large scale optoacoustic microscopy. Adv Sci. 2021;8(13):2004226. 10.1002/advs.202004226 PMC8261523 34258153

[44] Zhang HF, Maslov K, Stoica G, Wang LV. Functional photoacoustic microscopy for high-resolution and noninvasive in vivo imaging. Nat Biotechnol. 2006;24(7):848–851. 16823374 10.1038/nbt1220

[45] Park B, Oh D, Kim J, Kim C. Functional photoacoustic imaging: From nano- and micro- to macro-scale. Nano Converg. 2023;10(1):29. 37335405 10.1186/s40580-023-00377-3 PMC10279631

[46] Wang LV. Multiscale photoacoustic microscopy and computed tomography. Nat Photonics. 2009;3(9):503–509. 20161535 10.1038/nphoton.2009.157 PMC2802217

[47] Guo Z, Li L, Wang LV. On the speckle-free nature of photoacoustic tomography. Med Phys. 2009;36:4084–4088. 19810480 10.1118/1.3187231 PMC2738745

[48] Mantri Y, Jokerst JV. Impact of skin tone on photoacoustic oximetry and tools to minimize bias. Biomed Opt Express. 2022;13(2):875–887. 35284157 10.1364/BOE.450224 PMC8884230

[49] Fu L, Ling C, Jin Z, Luo J, Palma-Chavez J, Wu Z, Zhou J, Zhou J, Donovan B, Qi B, et al. Photoacoustic imaging of posterior periodontal pocket using a commercial hockey-stick transducer. J Biomed Opt. 2022;27(5):056005. 35610752 10.1117/1.JBO.27.5.056005 PMC9128833

[50] Lee C, Cho S, Lee D, Lee J, Park J-I, Kim H-J, Park SH, Choi W, Kim U, Kim C. Panoramic volumetric clinical handheld photoacoustic and ultrasound imaging. Photoacoustics. 2023;31: Article 100512. 37252650 10.1016/j.pacs.2023.100512 PMC10208888

[51] Zhang W, Ma H, Cheng Z, Wang Z, Xiong K, Yang S. High-speed dual-view photoacoustic imaging pen. Opt Lett. 2020;45(7):1599–1602. 32235952 10.1364/OL.388863

[52] Iversen M, Monisha M, Agarwala S. Flexible, wearable and fully-printed smart patch for pH and hydration sensing in wounds. Int J Bioprint. 2022;8(1):447. 35187277 10.18063/ijb.v8i1.447 PMC8852199

[53] Jankowska DA, Bannwarth MB, Schulenburg C, Faccio G, Maniura-Weber K, Rossi RM, Scherer L, Richter M, Boesel LF. Simultaneous detection of pH value and glucose concentrations for wound monitoring applications. Biosens Bioelectron. 2017;87:312–319. 27573297 10.1016/j.bios.2016.08.072

[54] Chen X, Wo F, Jin Y, Tan J, Lai Y, Wu J. Drug-porous silicon dual luminescent system for monitoring and inhibition of wound infection. ACS Nano. 2017;11(8):7938–7949. 28700206 10.1021/acsnano.7b02471

[55] Mokrý M, Gál P, Vidinský B, Kusnír J, Dubayová K, Mozes S, Sabo J. In vivo monitoring the changes of interstitial pH and FAD/NADH ratio by fluorescence spectroscopy in healing skin wounds. Photochem Photobiol. 2006;82(3):793–797. 16435883 10.1562/2005-09-08-RA-678

[56] Trengove NJ, Langton SR, Stacey MC. Biochemical analysis of wound fluid from nonhealing and healing chronic leg ulcers. Wound Repair Regen. 1996;4(2):234–239. 17177819 10.1046/j.1524-475X.1996.40211.x

[57] Mantri Y, Mishra A, Anderson CA, Jokerst JV. Photoacoustic imaging to monitor outcomes during hyperbaric oxygen therapy: Validation in a small cohort and case study in a bilateral chronic ischemic wound. Biomed Opt Express. 2022;13(11):5683–5694. 36733747 10.1364/BOE.472568 PMC9872873

[58] Han Z, Yuan M, Liu L, Zhang K, Zhao B, He B, Liang Y, Li F. pH-responsive wound dressings: Advances and prospects. Nanoscale Horiz. 2023;8(4):422–440. 36852666 10.1039/d2nh00574c

[59] Guo L, Zhang X, Zhao D-M, Chen S, Zhang W-X, Yu Y-L, Wang J-H. Portable photoacoustic analytical system combined with wearable hydrogel patch for pH monitoring in chronic wounds. Anal Chem. 2024;96(28):11595–11602. 38950152 10.1021/acs.analchem.4c02472

[60] Kamoun EA, Kenawy E-RS, Chen X. A review on polymeric hydrogel membranes for wound dressing applications: PVA-based hydrogel dressings. J Adv Res. 2017;8(3):217–233. 28239493 10.1016/j.jare.2017.01.005 PMC5315442

[61] Power G, Moore Z, O’Connor T. Measurement of pH, exudate composition and temperature in wound healing: A systematic review. J Wound Care. 2017;26(7):381–397. 28704150 10.12968/jowc.2017.26.7.381

[62] Jiang Y, Huang Y, Luo X, Wu J, Zong H, Shi L, Cheng R, Zhu Y, Jiang S, Lan L, et al. Neural stimulation in vitro and in vivo by photoacoustic nanotransducers. Matter. 2021;4(2):654–674.

[63] Jiang Y, Lee HJ, Lan L, Tseng H-a, Yang C, Man H-Y, Han X, Cheng J-X. Optoacoustic brain stimulation at submillimeter spatial precision. Nat Commun. 2020;11(1):881. 32060282 10.1038/s41467-020-14706-1 PMC7021819

[64] Zheng N, Fitzpatrick V, Cheng R, Shi L, Kaplan DL, Yang C. Photoacoustic carbon nanotubes embedded silk scaffolds for neural stimulation and regeneration. ACS Nano. 2022;16(2):2292–2305. 35098714 10.1021/acsnano.1c08491

[65] Han S, Park J, Choi WS, Youn I. Ultrasound stimulation increases neurite regeneration in injured dorsal root ganglion neurons through mammalian target of rapamycin activation. Brain Sci. 2020;10(7):409. 32629985 10.3390/brainsci10070409 PMC7407506

[66] Jiang W, Wang Y, Tang J, Peng J, Wang Y, Guo Q, Guo Z, Li P, Xiao B, Zhang J. Low-intensity pulsed ultrasound treatment improved the rate of autograft peripheral nerve regeneration in rat. Sci Rep. 2016;6:22773. 27102358 10.1038/srep22773 PMC4840319

[67] Du Z, Chen G, Li Y, Zheng N, Cheng J-X, Yang C. Photoacoustic: A versatile nongenetic method for high-precision neuromodulation. Acc Chem Res. 2024;57(11):1595–1607. 38759211 10.1021/acs.accounts.4c00119 PMC11154953

[68] Bueno FG, Moreira EA, de Morais GR, Pacheco IA, Baesso ML, Leite-Mello EV, de Palazzo JC. Enhanced cutaneous wound healing in vivo by standardized crude extract of Poincianella pluviosa. PLOS ONE. 2016;11(3): Article e0149223. 26938058 10.1371/journal.pone.0149223 PMC4777426

[69] Beckmann D, Lauckner G, Schmidt K, Asmussen B, Horstmann M, Koch A, Theobald F. Photoacoustic investigations on the penetration of drugs from transdermal therapeutic systems through human skin. Pharm Ind. 2002;64(3):271–277.

[70] Notingher I, Imhof RE. Mid-infrared in vivo depth-profiling of topical chemicals on skin. Skin Res Technol. 2004;10(2):113–121. 15059179 10.1111/j.1600-0846.2004.61.x

[71] Serizawa T, Onodera T, Oba K. Percutaneous absorption of a drug into hair follicles. Curr Probl Dermatol. 1995;22:195–200. 7587325 10.1159/000424253

[72] Sehn E, Silva KC, Retuci VS, Medina AN, Bento AC, Baesso ML, Storck A, Gesztesi JL. Photoacoustic spectroscopy to evaluate the penetration of sunscreens into human skin in vivo: A statistic treatment. Rev Sci Instrum. 2003;74(1):758–760.

[73] Rocha JCB, Pedrochi F, Hernandes L, de Mello JCP, Baesso ML. Ex vivo evaluation of the percutaneous penetration of proanthocyanidin extracts from Guazuma ulmifolia using photoacoustic spectroscopy. Anal Chim Acta. 2007;587(1):132–136. 17386764 10.1016/j.aca.2007.01.002

[74] Yamazaki M, Sato S, Ashida H, Saito D, Okada Y, Obara M. Measurement of burn depths in rats using multiwavelength photoacoustic depth profiling. J Biomed Opt. 2005;10(6): Article 064011. 16409076 10.1117/1.2137287

[75] Atiyeh BS, Gunn SW, Hayek SN. State of the art in burn treatment. World J Surg. 2005;29(2):131–148. 15654666 10.1007/s00268-004-1082-2

[76] Ida T, Kawaguchi Y, Kawauchi S, Iwaya K, Tsuda H, Saitoh D, Sato S, Iwai T. Real-time photoacoustic imaging system for burn diagnosis. J Biomed Opt. 2014;19(8): Article 086013. 25127338 10.1117/1.JBO.19.8.086013

[77] Todorović M, Jiao S, Ai J, Pereda-Cubián D, Stoica G, Wang LV. In vivo burn imaging using Mueller optical coherence tomography. Opt Express. 2008;16(14):10279–10284. 18607436 10.1364/oe.16.010279 PMC6986309

[78] Zhang HF, Maslov K, Stoica G, Wang LV. Imaging acute thermal burns by photoacoustic microscopy. J Biomed Opt. 2006;11(5): Article 054033. 17092182 10.1117/1.2355667

[79] Benavides-Lara J, Siegel AP, Tsoukas MM, Avanaki K. High-frequency photoacoustic and ultrasound imaging for skin evaluation: Pilot study for the assessment of a chemical burn. J Biophotonics. 2024;17(7): Article e202300460. 38719468 10.1002/jbio.202300460

[80] Wu Z, Duan F, Zhang J, Li S, Ma H, Nie L. In vivo dual-scale photoacoustic surveillance and assessment of burn healing. Biomed Opt Express. 2019;10(7):3425–3433. 31467787 10.1364/BOE.10.003425 PMC6706033

[81] Nam SY, Chung E, Suggs LJ, Emelianov SY. Combined ultrasound and photoacoustic imaging to noninvasively assess burn injury and selectively monitor a regenerative tissue-engineered construct. Tissue Eng Part C Methods. 2015;21(6):557–566. 25384558 10.1089/ten.tec.2014.0306 PMC4442601

[82] Coimbra R, Salim A, Diaz J Jr, Biffl WL, Winchell R, Napolitano L, Costantini T, Livingston DH, Inaba K. The journal of trauma and acute care surgery: Emergency general surgery algorithms article series. J Trauma Acute Care Surg. 2024;97(4):489. 39233333 10.1097/TA.0000000000004475

[83] Tsunoi Y, Sato N, Nishidate I, Ichihashi F, Saitoh D, Sato S. Burn depth assessment by dual-wavelength light emitting diodes-excited photoacoustic imaging in rats. Wound Repair Regen. 2023;31(1):69–76. 36177703 10.1111/wrr.13056

[84] Ida T, Iwazaki H, Kawaguchi Y, Kawauchi S, Ohkura T, Iwaya K, Tsuda H, Saitoh D, Sato S, Iwai T. Burn depth assessments by photoacoustic imaging and laser Doppler imaging. Wound Repair Regen. 2016;24(2):349–355. 26487320 10.1111/wrr.12374

[85] Xiong Y, Mi BB, Shahbazi MA, Xia T, Xiao J. Microenvironment-responsive nanomedicines: A promising direction for tissue regeneration. Mil Med Res. 2024;11(1):69. 39434177 10.1186/s40779-024-00573-0 PMC11492517

[86] Xiong Y, Mi B-B, Lin Z, Hu Y-Q, Yu L, Zha K-K, Panayi AC, Yu T, Chen L, Liu Z-P, et al. The role of the immune microenvironment in bone, cartilage, and soft tissue regeneration: From mechanism to therapeutic opportunity. Mil Med Res. 2022;9(1):65. 36401295 10.1186/s40779-022-00426-8 PMC9675067

[87] Hu X, Huang Y-Y, Wang Y, Wang X, Hamblin MR. Antimicrobial photodynamic therapy to control clinically relevant biofilm infections. Front Microbiol. 2018;9:1299. 29997579 10.3389/fmicb.2018.01299 PMC6030385

[88] Yaron JR, Gosangi M, Pallod S, Rege K. In situ light-activated materials for skin wound healing and repair: A narrative review. Bioeng Transl Med. 2024;9(3): Article e10637. 38818119 10.1002/btm2.10637 PMC11135152

[89] Maisch T. Resistance in antimicrobial photodynamic inactivation of bacteria. Photochem Photobiol Sci. 2015;14(8):1518–1526. 26098395 10.1039/c5pp00037h

[90] Hamblin MR, Hasan T. Photodynamic therapy: A new antimicrobial approach to infectious disease? Photochem Photobiol Sci. 2004;3(5):436–450. 15122361 10.1039/b311900a PMC3071049

[91] Feng Y, Liu L, Zhang J, Aslan H, Dong M. Photoactive antimicrobial nanomaterials. J Mater Chem B. 2017;5(44):8631–8652. 32264259 10.1039/c7tb01860f

[92] Rabiee N, Ahmadi S, Akhavan O, Luque R. Silver and gold nanoparticles for antimicrobial purposes against multi-drug resistance bacteria. Materials. 2022;15(5):1799. 35269031 10.3390/ma15051799 PMC8911831

[93] Zhang Z, Wang R, Xue H, Knoedler S, Geng Y, Liao Y, Alfertshofer M, Panayi AC, Ming J, Mi B, et al. Phototherapy techniques for the management of musculoskeletal disorders: Strategies and recent advances. Biomater Res. 2023;27(1):123. 38017585 10.1186/s40824-023-00458-8 PMC10685661

[94] Xie L, Pang X, Yan X, Dai Q, Lin H, Ye J, Cheng Y, Zhao Q, Ma X, Zhang X, et al. Photoacoustic imaging-trackable magnetic microswimmers for pathogenic bacterial infection treatment. ACS Nano. 2020;14(3):2880–2893. 32125820 10.1021/acsnano.9b06731

[95] Huang W, Hu B, Yuan Y, Fang H, Jiang J, Li Q, Zhuo Y, Yang X, Wei J, Wang X. Visible light-responsive selenium nanoparticles combined with sonodynamic therapy to promote wound healing. ACS Biomater Sci Eng. 2023;9(3):1341–1351. 36825832 10.1021/acsbiomaterials.2c01119

[96] Jeng S-F, Hsieh C-H, Lin T-S, Kuo Y-R, Wei F-C. Classification and reconstruction options in foot plantar skin avulsion injuries: Follow-up. Plast Reconstr Surg. 2003;112(6):220–221. 12832899 10.1097/01.PRS.0000066384.90361.5C

[97] Sakai G, Suzuki T, Hishikawa T, Shirai Y, Kurozumi T, Shindo M. Primary reattachment of avulsed skin flaps with negative pressure wound therapy in degloving injuries of the lower extremity. Injury. 2017;48(1):137–141. 27788928 10.1016/j.injury.2016.10.026

[98] Neuhaus K, Meuli M, Koenigs I, Schiestl C. Management of “difficult” wounds. Eur J Pediatr Surg. 2013;23(05):365–374. 24008551 10.1055/s-0033-1354588

[99] Zhang D, Yuan Y, Zhang H, Yi X, Xiao W, Yu A. Photoacoustic microscopy provides early prediction of tissue necrosis in skin avulsion injuries. Clin Cosmet Investig Dermatol. 2021;14:837–844. 10.2147/CCID.S316060 PMC8275181 34267532

[100] Hariri A, Chen F, Moore C, Jokerst JV. Noninvasive staging of pressure ulcers using photoacoustic imaging. Wound Repair Regen. 2019;27(5):488–496. 31301258 10.1111/wrr.12751 PMC8043767

[101] Huang C, Cheng Y, Zheng W, Bing RW, Zhang H, Komornicki I, Harris LM, Arany PR, Chakraborty S, Zhou Q, et al. Dual-scan photoacoustic tomography for the imaging of vascular structure on foot. IEEE Trans Ultrason Ferroelectr Freq Control. 2023;70(12):1703–1713. 37276111 10.1109/TUFFC.2023.3283139 PMC10809222

[102] Ivankovic I, Lin HA, Özbek A, Orive A, Deán-Ben XL, Razansky D. Multispectral optoacoustic tomography enables in vivo anatomical and functional assessment of human tendons. Adv Sci. 2024;11(18): Article e2308336. 10.1002/advs.202308336 PMC11095142 38445972

[103] Vionnet L, Gateau J, Schwarz M, Buehler A, Ermolayev V, Ntziachristos V. 24-MHz scanner for optoacoustic imaging of skin and burn. IEEE Trans Med Imaging. 2014;33(2):535–545. 24216682 10.1109/TMI.2013.2289930

[104] Jávor P, Rárosi F, Horváth T, Török L, Varga E, Hartmann P. Detection of exhaled methane levels for monitoring trauma-related haemorrhage following blunt trauma: Study protocol for a prospective observational study. BMJ Open. 2022;12(7): Article e057872. 10.1136/bmjopen-2021-057872 PMC9260765 35793921

[105] Chen Y-S, Frey W, Kim S, Kruizinga P, Homan K, Emelianov S. Silica-coated gold nanorods as photoacoustic signal nanoamplifiers. Nano Lett. 2011;11(2):348–354. 21244082 10.1021/nl1042006 PMC3040682

[106] Kang B, Yu D, Dai Y, Chang S, Chen D, Ding Y. Cancer-cell targeting and photoacoustic therapy using carbon nanotubes as “bomb” agents. Small. 2009;5(11):1292–1301. 19274646 10.1002/smll.200801820

[107] Agarwal A, Huang SW, O’Donnell M, Day KC, Day M, Kotov N, Ashkenazi S. Targeted gold nanorod contrast agent for prostate cancer detection by photoacoustic imaging. J Appl Phys. 2007;102(6): Article 064701.

[108] Moon H, Kumar D, Kim H, Sim C, Chang J-H, Kim J-M, Kim H, Lim DK. Amplified photoacoustic performance and enhanced photothermal stability of reduced graphene oxide coated gold nanorods for sensitive photoacoustic imaging. ACS Nano. 2015;9(3):2711–2719. 25751167 10.1021/nn506516p

[109] Sheng Z, Song L, Zheng J, Hu D, He M, Zheng M, Gao G, Gong P, Zhang P, Ma Y, et al. Protein-assisted fabrication of nano-reduced graphene oxide for combined in vivo photoacoustic imaging and photothermal therapy. Biomaterials. 2013;34(21):5236–5243. 23602365 10.1016/j.biomaterials.2013.03.090

[110] Zha Z, Deng Z, Li Y, Li C, Wang J, Wang S, Qu E, Dai Z. Biocompatible polypyrrole nanoparticles as a novel organic photoacoustic contrast agent for deep tissue imaging. Nanoscale. 2013;5(10):4462–4467. 23584573 10.1039/c3nr00627a

[111] Pu K, Chattopadhyay N, Rao J. Recent advances of semiconducting polymer nanoparticles in in vivo molecular imaging. J Control Release. 2016;240:312–322. 26773769 10.1016/j.jconrel.2016.01.004 PMC4938792

[112] Bettinger CJ, Cyr KM, Matsumoto A, Langer R, Borenstein JT, Kaplan DL. Silk fibroin microfluidic devices. Adv Mater. 2007;19(5):2847–2850. 19424448 10.1002/adma.200602487 PMC2677821

[113] Won Baac H, Ok JG, Park HJ, Ling T, Chen S-L, Hart AJ, Guo LJ. Carbon nanotube composite optoacoustic transmitters for strong and high frequency ultrasound generation. Appl Phys Lett. 2010;97(23): Article 234104. 21200445 10.1063/1.3522833 PMC3013153

[114] Brown JE, Diaz L, Christoff-Tempesta T, Nesbitt KM, Reed-Betts J, Sanchez J, Davies KW. Characterization of nitrazine yellow as a photoacoustically active pH reporter molecule. Anal Chem. 2015;87(7):3623–3630. 25741857 10.1021/ac503515k

[115] Dong H, Wang L, Du L, Wang X, Li Q, Wang X, Zhang J, Nie J, Ma G. Smart polycationic hydrogel dressing for dynamic wound healing. Small. 2022;18(25): Article e2201620. 35599229 10.1002/smll.202201620

[116] Liang Y, He J, Guo B. Functional hydrogels as wound dressing to enhance wound healing. ACS Nano. 2021;15(8):12687–12722. 34374515 10.1021/acsnano.1c04206

[117] Li J, Esteban-Fernández de Ávila B, Gao W, Zhang L, Wang J. Micro/nanorobots for biomedicine: Delivery, surgery, sensing, and detoxification. Sci Robot. 2017;2(4):eaam6431. 31552379 10.1126/scirobotics.aam6431 PMC6759331

[118] Nelson BJ, Kaliakatsos IK, Abbott JJ. Microrobots for minimally invasive medicine. Annu Rev Biomed Eng. 2010;12:55–85. 20415589 10.1146/annurev-bioeng-010510-103409

[119] Lee H, Dellatore SM, Miller WM, Messersmith PB. Mussel-inspired surface chemistry for multifunctional coatings. Science. 2007;318(5849):426–430. 17947576 10.1126/science.1147241 PMC2601629

[120] Li X, Jiang M, Zeng S, Liu H. Polydopamine coated multifunctional lanthanide theranostic agent for vascular malformation and tumor vessel imaging beyond 1500 nm and imaging-guided photothermal therapy. Theranostics. 2019;9(13):3866–3878. 31281519 10.7150/thno.31864 PMC6587345

[121] Hong S, Kim KY, Wook HJ, Park SY, Lee KD, Lee DY, Lee H. Attenuation of the in vivo toxicity of biomaterials by polydopamine surface modification. Nanomedicine. 2011;6(5):793–801. 21793672 10.2217/nnm.11.76

[122] Medina-Sánchez M, Schmidt OG. Medical microbots need better imaging and control. Nature. 2017;545(7655):406–408. 28541344 10.1038/545406a

[123] Zhu H, Fang Y, Miao Q, Qi X, Ding D, Chen P, Pu K. Regulating near-infrared photodynamic properties of semiconducting polymer nanotheranostics for optimized cancer therapy. ACS Nano. 2017;11(9):8998–9009. 28841279 10.1021/acsnano.7b03507

[124] Zheng D, Yu P, Wei Z, Zhong C, Wu M, Liu X. RBC membrane camouflaged semiconducting polymer nanoparticles for near-infrared photoacoustic imaging and photothermal therapy. Nanomicro Lett. 2020;12(1):94. 34138120 10.1007/s40820-020-00429-x PMC7770914

[125] Li J, Pu K. Development of organic semiconducting materials for deep-tissue optical imaging, phototherapy and photoactivation. Chem Soc Rev. 2019;48(1):38–71. 30387803 10.1039/c8cs00001h

[126] Valluru KS, Wilson KE, Willmann JK. Photoacoustic imaging in oncology: Translational preclinical and early clinical experience. Radiology. 2016;280(2):332–349. 27429141 10.1148/radiol.16151414 PMC4976462

[127] Wang X, Ren X, Yang J, Zhao Z, Zhang X, Yang F, Zhang Z, Chen P, Li L, Zhang R. Mn-single-atom nano-multizyme enabled NIR-II photoacoustically monitored, photothermally enhanced ROS storm for combined cancer therapy. Biomater Res. 2023;27:125. 38049922 10.1186/s40824-023-00464-w PMC10694968

