# Supplementary material for: The Potential of Photoacoustic Imaging in Detecting and Managing Complex Wounds
Source: Biomater Res. 2025 May 21;29:0206. doi: 10.34133/bmr.0206 (PMC12092969; doi:10.34133/bmr.0206)
Supplement: Supplementary 1 — Fig. S1 [file bmr.0206.f1.zip › Supplementary materials.docx]

**The Potential of Photoacoustic Imaging in Detecting and Managing Complex Wounds**

**Authors**

Haifeng Hu^†,1^,Ruiyin zeng^†,1^,Longyu Du^1^,Weixian Hu^1^,Chuanlu Lin^1^,Jiewen Liao^1^,Chong Ding^1^,Xudong Xie^1^,Bobin Mi^1^,Wu Zhou^*^,Yun Sun^*^,Faqi Cao^*^,Guohui Liu^*, 1^.

[1] Department of Orthopedics, Union Hospital, Tongji Medical College, Huazhong University of Science and Technology, Wuhan 430022, China.

^†^ These authors contributed equally to this article.

^*^Address correspondence to: liuguohui@hust.edu.cn (G. Liu); 13971293030@qq.com (F. Cao); 627224540@qq.com (Y. Sun);2016XH0120@hust.edu.cn(W.Zhou)

**Supplemental Figure 1**

**
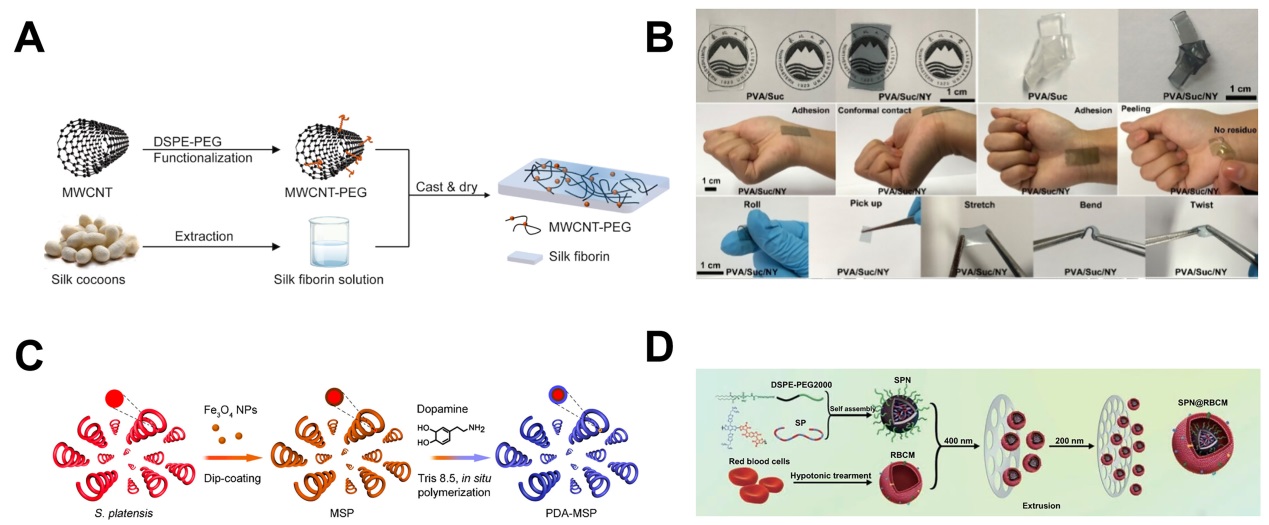
**

**Fig.S1. Fabrication diagram of multiple materials.** (A) Illustration of the CNT/silk fabrication process. (B)The transmittance, knotting ability, skin adhesion, and flexibility of both PVA/Suc and PVA/Suc/NY hydrogels are discussed. (C) A schematic illustrates the fabrication process of PDA-MSP derived from Spirulina platensis (D)Schematic depiction of SPN@RBCM nanoparticle preparation. Figures are reproduced with permission from [60], © 2024, American Chemical Society. Figures reprinted with permission from [65], © 2022 American Chemical Society. Figures reproduced with permission from [95]. © 2020 American Chemical Society. reproduced under Creative Commons Attribution 4.0 International License [125], © The Author(s) 2020.
