# Supplementary figures and images for: The Potential of Photoacoustic Imaging in Detecting and Managing Complex Wounds
Source: Biomater Res. 2025 May 21;29:0206. doi: 10.34133/bmr.0206 (PMC12092969; doi:10.34133/bmr.0206)

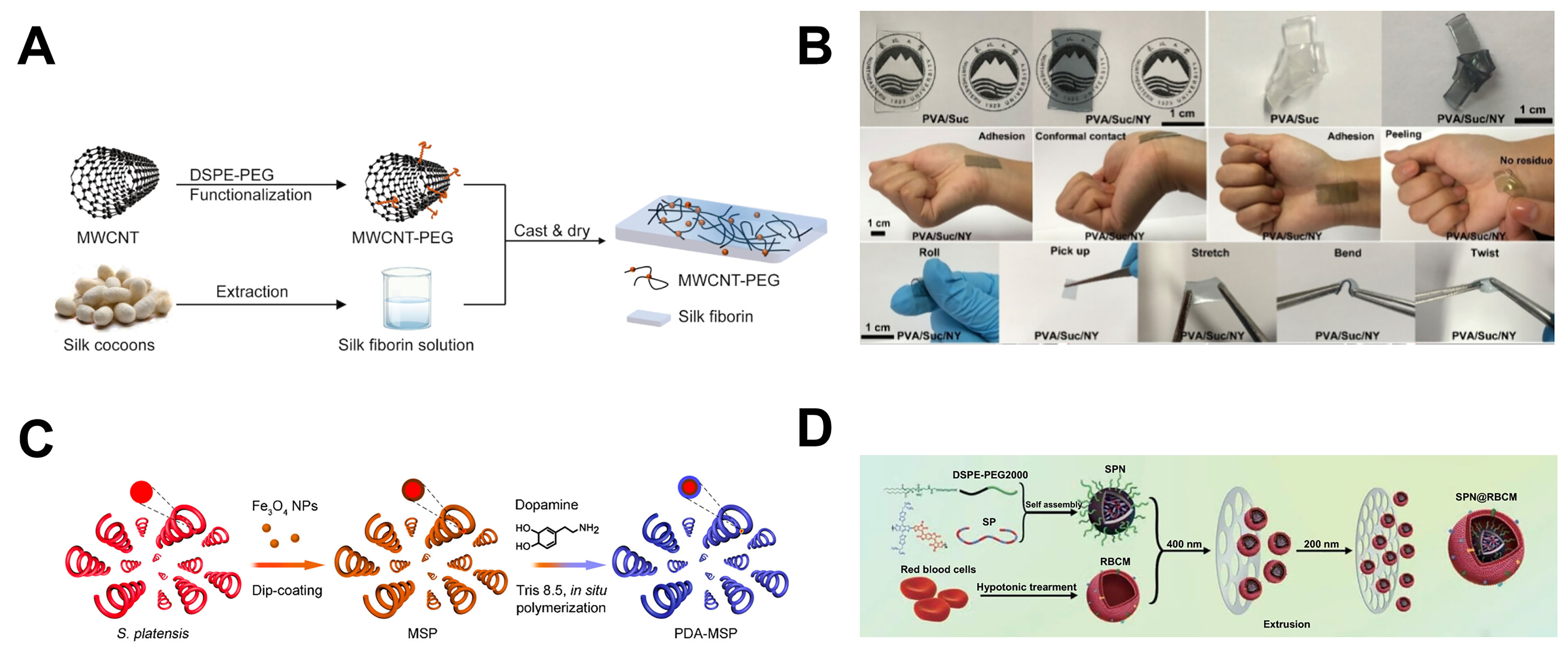

Supplement: Supplementary 1 — Fig. S1 [file bmr.0206.f1.zip › Figure supplement 1.jpg]
